# Interactive medical and safety monitoring in clinical trials with clinDataReview: a validated and open-source reporting tool

**DOI:** 10.3389/fmed.2024.1356323

**Published:** 2024-07-11

**Authors:** Laure Cougnaud, Margaux Faes, Dirk Van Krunckelsven, Arne De Roeck, Michela Pasetto, Ann Fieuw, Claus A. Andersen, Paul Meyvisch

**Affiliations:** ^1^Open Analytics NV, Antwerp, Belgium; ^2^Galapagos NV, Mechelen, Belgium

**Keywords:** clinical data, safety monitoring, medical monitoring, R package validation, clinical trial, clinDataReview R package

## Abstract

Continuous medical and safety monitoring of subject data during a clinical trial is a critical part of evaluating the safety of trial participants and as such is governed by protocol procedures and regulatory guidelines to meet the trial's intended objectives. We present an open-source validated graphical tool (clinDataReview R package) which provides access to the trial data with drill-down to individual patient profiles. The tool incorporates functionalities that facilitate detection of error and data inconsistencies requiring follow-up. It supports regular medical monitoring and oversight as well as safety monitoring committees with interactive tables and listings alongside graphical visualizations of the primary safety data in reports. An implementation example is given where the tool is used to deliver validated outputs following FDA/EMA guidelines. As such, this tool enables a more efficient, interactive, and reproducible review of safety data collected during an ongoing clinical trial.

## Introduction

Monitoring of clinical trials is performed for a variety of reasons. Clinical monitoring is done to oversee the progress of a study to ensure it is conducted in accordance with the protocol. Medical monitoring provides general support to clinical monitors and medical site staff and consists of review of eligibility criteria, critical laboratory values, general safety matters, use of medication etc. Both clinical and medical monitoring is the responsibility of the study sponsor and may or may not be outsourced to a service provider.

The safety and overall benefit-risk of the trial participants is commonly overseen by the sponsor and external (possibly Independent) Data (or Safety) Monitoring Committees or (I)DMC (or SMC). Similar to the clinical and medical monitoring, this monitoring effort can be conducted in a blinded or unblinded fashion depending on the study phase and trial integrity measures.

Each of the individuals and committees, internal or external to the sponsor, tasked with monitoring of the clinical trial are required to have continuous or regular access to the trial safety data, either raw data, individual or aggregate data, which poses a considerable operational challenge to the study sponsor. This challenge can be addressed in numerous ways which may or may not include outsourcing of certain activities.

Traditionally, safety data can be delivered in the form of static Tables, Listings, and Figures (TLFs). While these are useful for standardized high-level safety reporting for submission to the regulatory authorities, they lack the interactivity needed to drill down into safety signals, to explore all safety readouts on a patient-level, review aggregate data, ensure signal detection or trending.

Hence, interactive medical and safety monitoring systems—which enable the user to select specific patients, combine different datasets of interest and interact with the data visualizations—are gaining popularity. Among these systems, commercial solutions such as JReview^1^ and Spotfire dashboards^2^ stand out. These kinds of systems offer dynamic visualizations and real-time analysis, empowering non-technical users (researchers and clinicians) to make informed decisions swiftly. Both tools offer extensive customization options, allowing users to tailor visualizations, reports, and analyses according to specific trial requirements. While they are both widely used across the life sciences industry, they pose some drawbacks specifically for smaller organizations. The substantial cost associated with these systems can pose a significant barrier for organizations with limited financial resources. Their effective utilization relies on the availability of in-house clinical databases. Organizations lacking this data infrastructure, such as those working with outsourced models, may not be able to harness the potential of these systems. The real-time analysis of data might not allow trace back of preceding visualizations that were used for decision making, whereas the importance of the traceability and documentation in decision-making processes related to safety monitoring is emphasized within the ICH E6 (R2) Good Clinical Practice guideline (1). Moreover, these tools may also lead to inconsistent safety reporting due to individual reviewer customization, can be error prone, may become too complex (requiring training), and are frequently only available to selected internal staff (as supported by the chosen license model).

Free, open-source alternatives such as the Safety Explorer Suite (2), via the safetyGraphics R package have been introduced in recent years. This tool is designed around the Clinical Data Interchange Standards Consortium's (CDISC's)^3^ Analysis Data Model (ADaM)^4^ standard (but also supports non-standard data), which means the charts use datasets which may only be available at certain regular intervals and, in contrast to JReview or Spotfire, not on a real-time basis. The Safety Explorer Suite consists of a set of standard interactive graphics and tables, accessible via a Shiny (3) application, created to monitor key safety metrics. Each graphic created in the application (and code to produce it) can be exported from the application, which supports the reproducibility of the output. However, this alternative currently still lacks desirable displays that go beyond safety data, such as baseline comparison of demographics, medical history, concomitant medication, and operational characteristics of the trial which would be essential for the purpose of a comprehensive safety evaluation and does not offer a fully traceable workflow.

To address these issues, an interactive open-source reporting tool for safety and medical monitoring (clinDataReview R package) is developed to help the medical monitors and (I)DMCs with the exploration of (any standard) clinical data. This tool creates a single, clearly structured, modular medical monitoring report combining interactive summary tables, listings, and visualizations of safety and non-safety data, linked with patient profiles. The tool enables the standardization of the reporting of the clinical data across studies, while being flexible enough to be tailored to the study of interest. The delivered report is standalone, enhancing traceability of the entire output, and thus easily shareable among all involved parties.

Similarly to the development of the safetyGraphics tool, the development of this tool was an interdisciplinary work. The graphs were developed in collaboration with clinicians and statisticians to construct an interactive tool that facilitates and efficient reviewer workflow.

This article will provide an overview of the software and methodology and an example of the GxP compliant implementation of this tool at a biotechnology company. Thanks to the quality-controlled software and infrastructure implemented, the report is fully reproducible, traceable, and archivable, following the FDA 21 CFR Part 11 regulation (4). Furthermore, a Continuous Integration/Continuous Development system has been set up with automated validation of the tool, which allows improvements to the open-source tool and correction turnaround times to be greatly reduced.

## Materials and methods

The clinical data review tool allows users to produce standalone reports, containing interactive TLFs for the data collected during a trial. In this section, the different components of the tool are introduced. We will discuss the clinDataReview tool in four sections: (i) Input data, (ii) Tool components, (iii) Set up of the tool, (iv) Qualification/validation.

### Input data

The clinical data review tool supports clinical data in any tabular long format, including (but not limited to) CDISC's Standard Data Tabulation Model (SDTM)^5^ and ADaM.

The clinical study data is collected through the electronic Case Report Forms (eCRF) from local study sites. The eCRFs are then collated into an SDTM compliant dataset and automatically transferred. Regular snapshots of the clinical data base, delivering data at fixed time intervals during the clinical trial support the frequent exploration and detection of any safety events as early as possible. The data is stored in a separate validated data repository in a standardized folder structure (by study and batch), with versioning in place. In double-blind trials, data is delivered blinded by default. For (I)DMC/SMC where unblinded information is needed, the unblinding and the storage of the unblinded data and report are performed according to company's GCP compliant procedures. At the biotechnology company, the direct input data for the tool are usually very light analysis datasets built from the SDTM and inspired by CDISC's ADaM, to limit the data pre-processing but still facilitate the data exploration. Simple derivations are performed such as the fine-tuning of timing variables (derivation of relative days, or data flags to assess missing dates for the visualization of time profiles), the creation of ordering variables (numeric severity for adverse event severity), the derivation of categorical variables from continuous variables for summary tables (e.g., age categorization), or the creation of analysis flags if required. The most complex derivations performed are the possible averaging of measurements of the same visit (within a time window) in the Electrocardiogram (EG) and Vital Signs (VS) domain and creation of QTcF value in the EG domain if its constituent measurements exist. This data-handling relies heavily on a solid SDTM implementation, and as such also incorporates data checks (mostly existence of variables).

### Tool components

The clinical data review tool consists of a report with interactive TLFs linked to individual patient profiles that can be tailored to the specific clinical trial at hand. The entire interactive report, TLFs, patient profiles and in-text tables are created via the combination of several standalone open-source R (5) packages.

### Patient profiles

The patient profile report displays an overview of the information for a specific trial patient (demography, treatment exposure, adverse event, laboratory measurements, …) in a static pdf report. This report consists of multiple visualization modules: text, event, interval, and line that can be tailored to the datasets and variables of interest. The patient profiles are created with the patientProfilesVis R package.

### In-text tables

Summary tables of descriptive statistics of domains and metrics of interest in the clinical trial are available in the report. Tables are available in an interactive format to enhance data exploration (filtering, ordering) and in a static (MSOffice Word) format, to align with standard Clinical Study Report format, for medical reporting purposes. The summary tables are created with the inTextSummaryTable R package.

### Interactive report and TLFs

The interactive visualizations and reports are created with the clinDataReview R package. The report consists of a series of standalone HTML documents containing interactive TLFs and patient profiles. The tables (and listings) and patient profiles are created with the previously cited R packages.

Each interactive TLF provides a high-level view of specific data of interest. Each visualization is coupled with an interactive table containing the data behind the visualization, such that the medical monitor can further explore signals of interest by exporting a subset of the data of interest in their preferred file format (Excel, csv, PDF).

The figures and tables support the display of individual cases (e.g., listings, and subject-specific line plots and scatterplots) and aggregated data across patients (e.g., summary tables or figures). From each of these summary views, when a subject or signal of interest is detected, the information can be drilled down to subject level information, via hover/click on visualizations, expandable content in tables and hyperlinks in listings via the subject profile. As changes between consecutive data transfers are typically of interest, summary statistics or listings of the encountered differences can be compared between two data batches. The report files can be easily stored and archived within existing IT infrastructure for traceability.

Reports generated on blinded or unblinded data only differ by the inclusion of the treatment variable for the separated computation of summary statistics or indication in visualizations or listings.

### Set up of the tool

**Figure 1:**
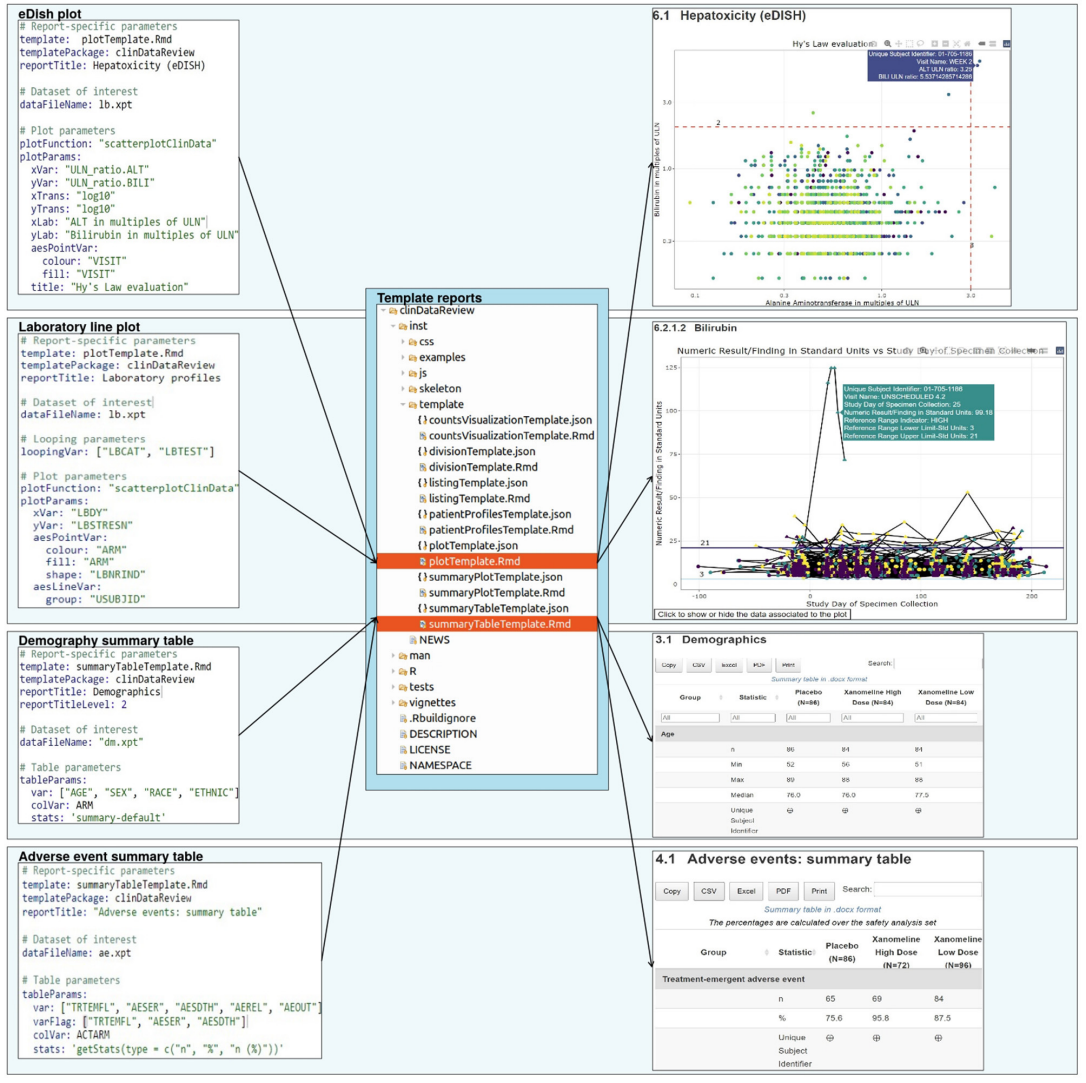
Report creation: a set of configuration files, each associated to a template report, specify the design for the report.

The clinical data review report is configured by a set of configuration files and generic template reports (Figure 1).

### Configuration files

The user can customize the report for a specific clinical trial or dataset of interest via the use of configuration files, which requires minimal R expertise. The configuration file contains the input parameters for each chapter of the report, such as the analysis dataset (domain), variables of interest within the dataset, type of output to be created (visualizations, tables, listing), (limited) data formatting, data location, date and study information.

This enables an efficient separation of a (generic) code generating the report and the trial-specific settings. The configuration files are available in a human-readable format [YAML^6^] which facilitates the transfer of information for users not familiar with the back-end technology and ensures the transparent data selection and processing steps. A set of default configuration files (for blinded and unblinded data) is available in the clinDataReview package and can be extended or customized to support the creation of a standard report for a new study.

### Template reports

**Figure 2:**
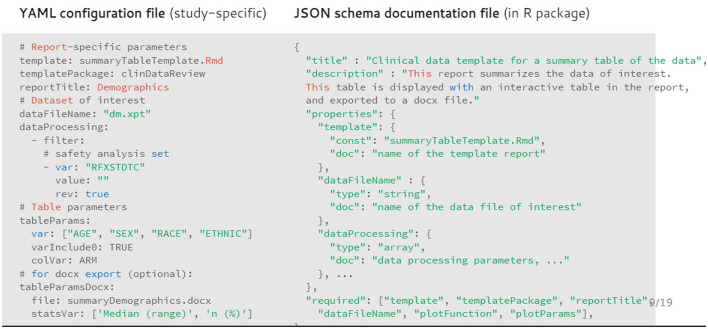
Example of configuration file (in YAML format) for the demography summary table and associated documentation file (in JSON format).

The report is generated from template (R Markdown) reports, to follow the literate programming paradigm (6). Each template is associated to a JSON schema file containing the description of the content of the template and associated input parameters. This file enables the creation of the template documentation in standard R documentation (Rd) format during the R package creation, and the checking of the input parameters from the YAML configuration file (for the presence and type) during the report creation (Figure 2).

The template and associated JSON specification file of the input parameters are stored inside the R package as well. Each chapter of the report is rendered in separate R sessions and stored as a standalone HTML page to ensure faster report loading in the browser for viewing data from larger trials. Because of the modular implementation of the tool, any in-text table, patient profile visualization, interactive visualization, or chapter of the report of interest can be created outside of the clinical data review report.

## Qualification/validation: an industry example

### Qualification of software

At the biotechnology company, qualification of the R programming language is performed according to the guideline of the R foundation board (7) including a full performance qualification on the production system using the more than thousand unit tests provided.

This production system consists of Docker (8) containers running on a Kubernetes^7^ server with in-house repositories [Elastic Container Registry^8^ for Docker images, CodeCommit^9^ git for code, RDepot^10^ for R packages and Nexus^11^ for external system dependencies] to ensure full reproducibility within an Infrastructure-as-Code setup. When reports are created using the tool, they are created in a reproducible & isolated environment (via Docker), ensuring a fixed version of the code, R packages and system dependencies. Installation, operation, and performance qualification tests are executed to show that the production system satisfies the necessary requirements to ensure proper functioning of the tool.

The reproducibility and retrieval of the report is ensured via the use of version control tools. Git^12^ is used for the storing of the code (including the R packages) that creates the report. SAS Institute's LSAF^13^ is used for storing the study-specific data, configuration files and report. In this data warehouse, user access and protection against unauthorized access or modifications of the data and report are controlled, ensuring the security and integrity of the electronic records. Secure, computer-generated, time-stamped audit trails are stored in the data warehouse to track actions performed by the computerized systems on these records. This ensures compliance with the FDA 21 CFR Part 11 regulation (4).

### Validation of tool and output

As part of the validation of the tool for GxP usage, the suite of R packages is covered by unit tests (Table 1). The tests are included inside each R package. Each update of the R packages is followed by an automated execution of the old and new unit tests on the Continuous Integration/Continuous Development platform Jenkins^14^ and the results are exported into a verification document, which subsequently is reviewed by the Quality Assurance representative before release of the new version.

**Table 1:** Summary of the unit tests and code coverage for each of the R package.

**R package**	**Number of unit tests**	**Line coverage**
clinUtils (v0.1.4)	338	99%
clinDataReview (v1.4.0)	606	98%
inTextSummaryTable (v3.3.0)	996	99%
patientProfilesVis (v2.0.5)	464	98%

The clinUtils R package contains general utility functions for use across the different R packages.

This ensures that all computer software used in the statistical analysis (R and the tool-specific R packages) is reliable, and that the documentation of the testing procedure is available as mandated by the ICH E9 guideline (9).

The data handling from SDTM to light analysis data sets is performed by a suite of SAS macros and template programs which have been validated by a series of tests scripts, re-run with each new release of the suite of programs.

## Results

In clinical trials, the frequent review of safety data collected on patients is a key process to safeguard patient safety. To conduct proper safety monitoring, the medical monitors need a good view of the safety information collected throughout the trial, to spot trends and rapidly detect changes in the safety data. We have developed a novel tool with interactive summary tables, listings, and visualizations of safety data, linked with patient profiles.

A typical user journey of the review of clinical data with the clinical data review tool is outlined below using a few highlighted examples. A complete example report is available at: https://medical-monitoring.openanalytics.io/.

### Subject disposition

**Figure 3:**
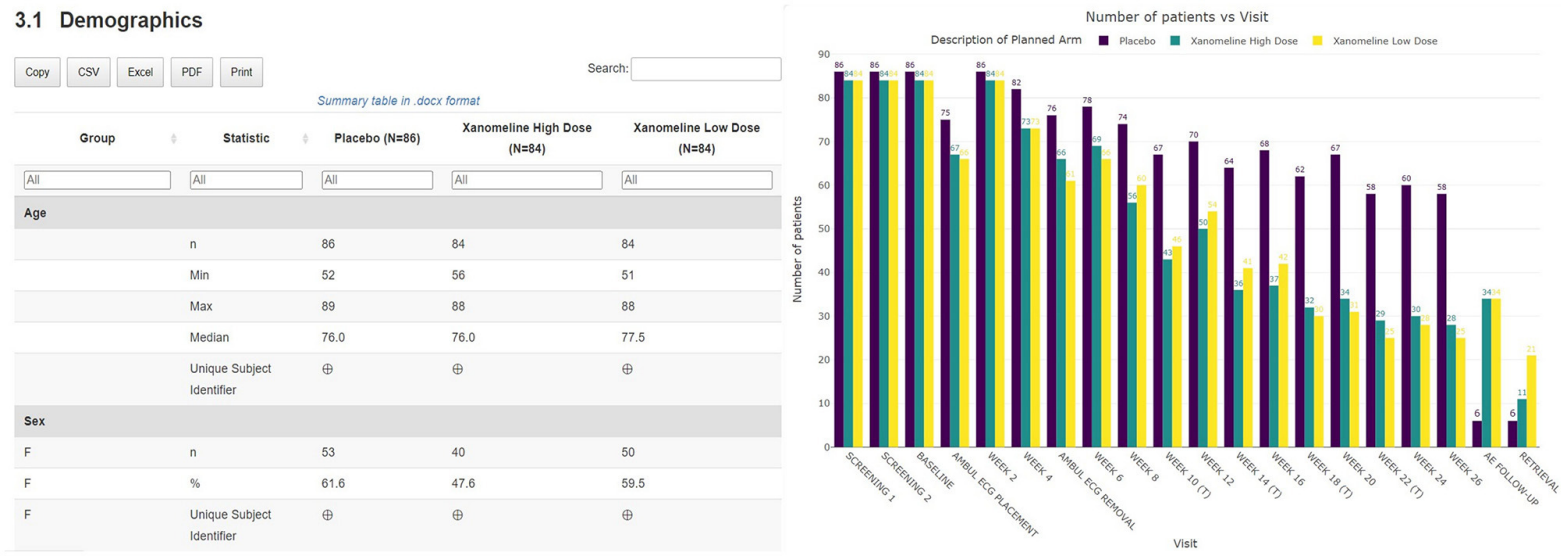
Summary table of demographic characteristics and barplot visualization of the number of patients by visit by treatment arm.

A thorough safety review starts with a good understanding of the study population. The clinical data review report tool provides an overview of the general subject disposition, which includes the number of subjects in the study split by demographic characteristics (as well as by treatment for unblinded data). The recruitment and visit attendance are checked via the visualization of the number of patients by visit (Figure 3). This study snapshot bar chart can be a very helpful tool to present the number of participants that were randomized and who attended each visit. This visualization clearly shows how much data are available at later study visits relative to the number randomized in the study. A subject disposition table provides counts and percentage of discontinuations, while reasons for discontinuation are captured in the associated listing. From these views, the reviewer is able to examine the discontinuation rate in the different treatment arms. This serves as the starting point to explore the safety of the patients.

### Adverse events

**Figure 4:**
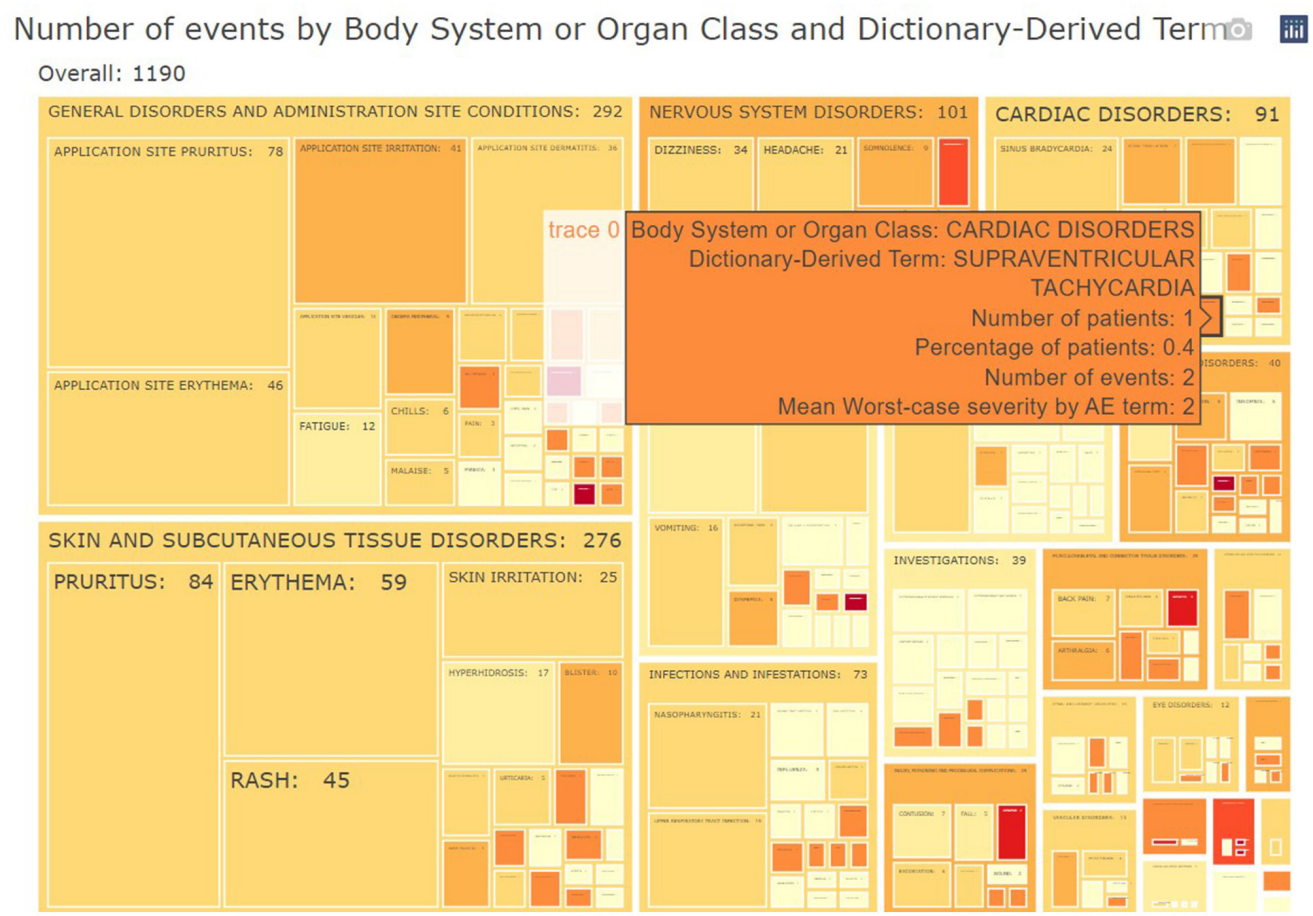
Example of aggregated data across patients: treemap of adverse events. For each group (Body System or Organ Class) of adverse events, statistics of interest (number/percentages of subjects, number of events and severity) are displayed.

**Figure 5:**
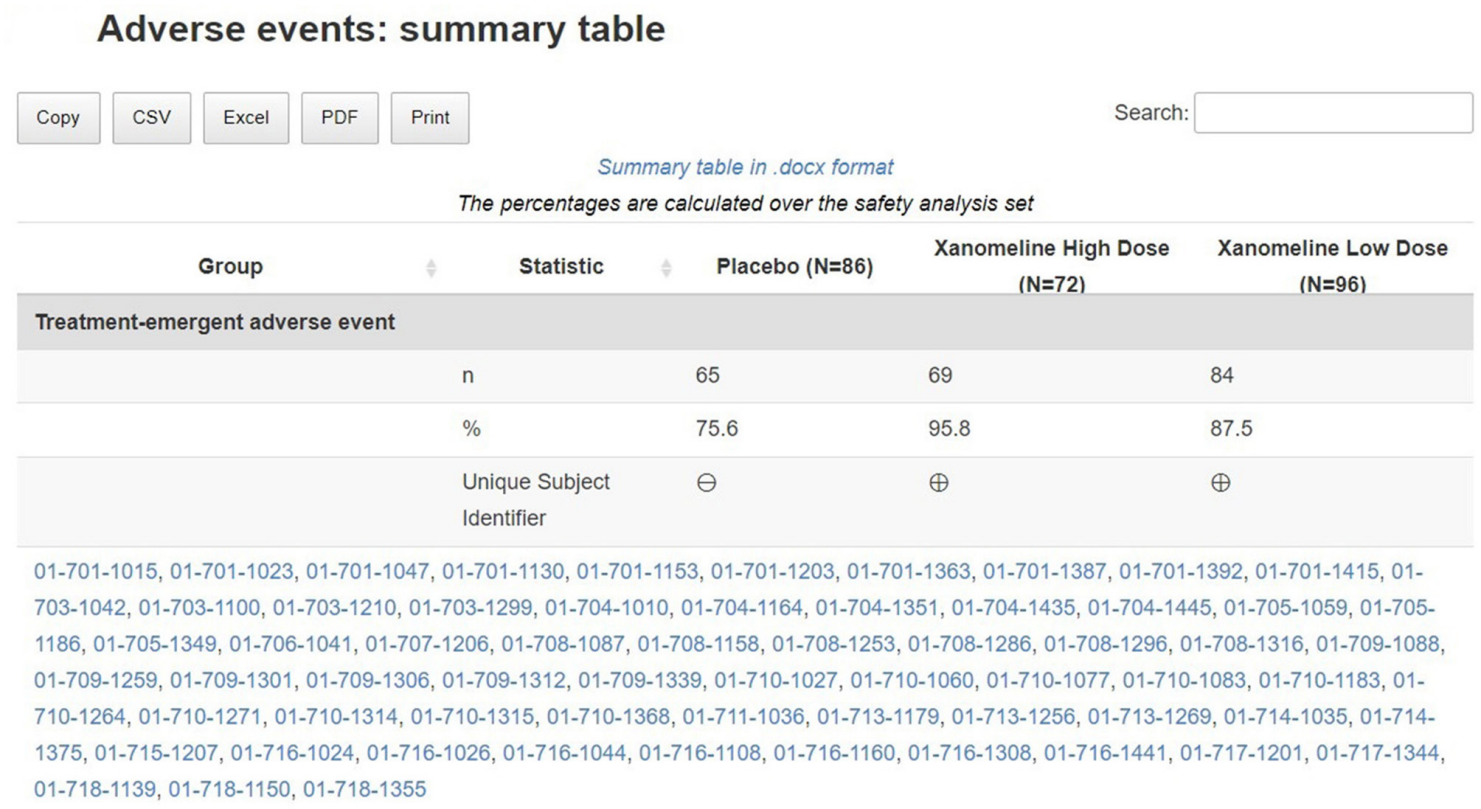
Link between aggregated and individual patient level: summary table of treatment-emergent adverse events. For each treatment, subjects presenting such adverse events are listed in a collapsible cell of the table and linked to the patient profile.

The subject disposition is then followed by a general picture of the adverse events in the study population. The medical monitor can evaluate the adverse events, serious adverse events, adverse events of interest (if specified) aggregated across patients. The tool provides treemap and sunburst visualizations (Figure 4) and summary tables (Figure 5). This allows for a high-level evaluation of the most frequent adverse events, their severity, or relatedness to drug exposure. The treemap visualizations can be configured such that they display the worst grade for each adverse event term or body system with different types of shading. If any safety imbalance across treatments is detected in the summary table, the medical monitor can easily navigate back to the subject disposition section to investigate if this finding stems from a demographic or disease characteristics imbalance at baseline or differences in exposure time. All adverse events in the listings can be filtered, for example for seriousness, severity, relatedness to drug, and outcome. Any adverse events (especially those of special interest) can be further investigated on a patient-level. The corresponding subjects are listed, and the medical monitor is directed through a hyperlink to the patient profile of this subject.

### Comparison between data batches

**Figure 6:**
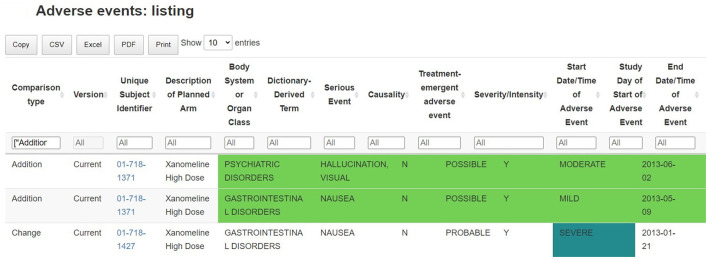
Identification of new safety events: listing of adverse events, with comparison between successive data batches. Records (and variables) which are new, removed or changed in the new data batch are highlighted in the table.

A key feature of the tables and listings in the adverse events section is that they have been developed to assist the medical monitor in answering the question “what has changed since the last review of the data” by providing a side-by-side comparison of the summary statistics of the previous data batch in the tables and flagging new adverse events occurring since the latest data collection date in the listings. The interactive listings enable the comparison of records between successive data batches, highlighting new safety events (flagged as additions) and changes to earlier information of interest. Just like the interactive tables, the listing can be exported to other file formats, which allows the medical monitor to further process the data with other software such as Excel, or Word (albeit outside GxP control) (Figure 6).

Such listing supports the ongoing quality review of the incomplete and potentially inaccurate data throughout the trial: any adverse events that are not correctly filled in, obvious incorrect dates or other data-related issue are flagged and can be linked to the clinical site information. This allows for a continuous data review and follow-up, where updates are clearly flagged between previous and current data releases.

### Signal detection

**Figure 7:**
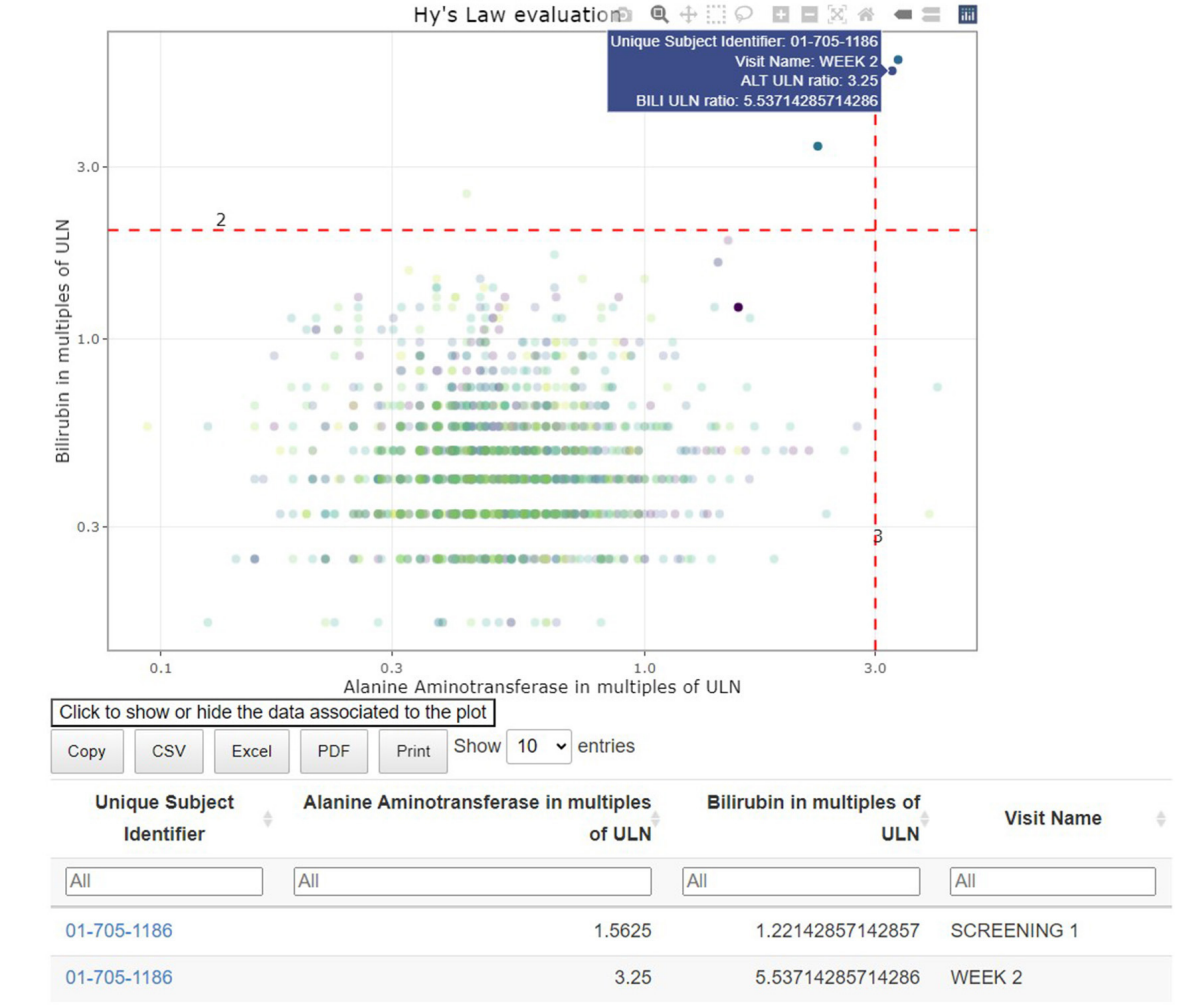
Link between aggregated and individual patient data: hepatotoxicity eDish visualization. An outlying measurement is selected in the visualization, which highlights all visits of the same patient in the visualization and the attached table.

The interactive visualizations are useful in signal and trend detection. Continuous safety parameters such as laboratory values are visualized using patient-level spaghetti plots, aggregated line plots or shift plots. These visualizations help detect trends over time for all patients by treatment arm. Lines on the graphs, reflecting the abnormality thresholds for the specific parameters, are added to assist in efficient detection of abnormal values. As with all tables and figures, configuration can be updated to show the trajectories of a narrower subset of patients such as those subjects with at least one abnormal laboratory value. As such, the medical monitor can easily identify an unexpected value of a parameter (as highlighted in Figure 7), where the data from the subject in the right-hand corner has been selected. Selecting a patient of interest in these graphs will highlight the actual values of this subject at different visits for the medical monitor and will display those in the associated table below. If needed the drill down to the patient level in the patient profile can be used next to better understand the occurrence of this unusual value.

### Individual patient overview

**Figure 8:**
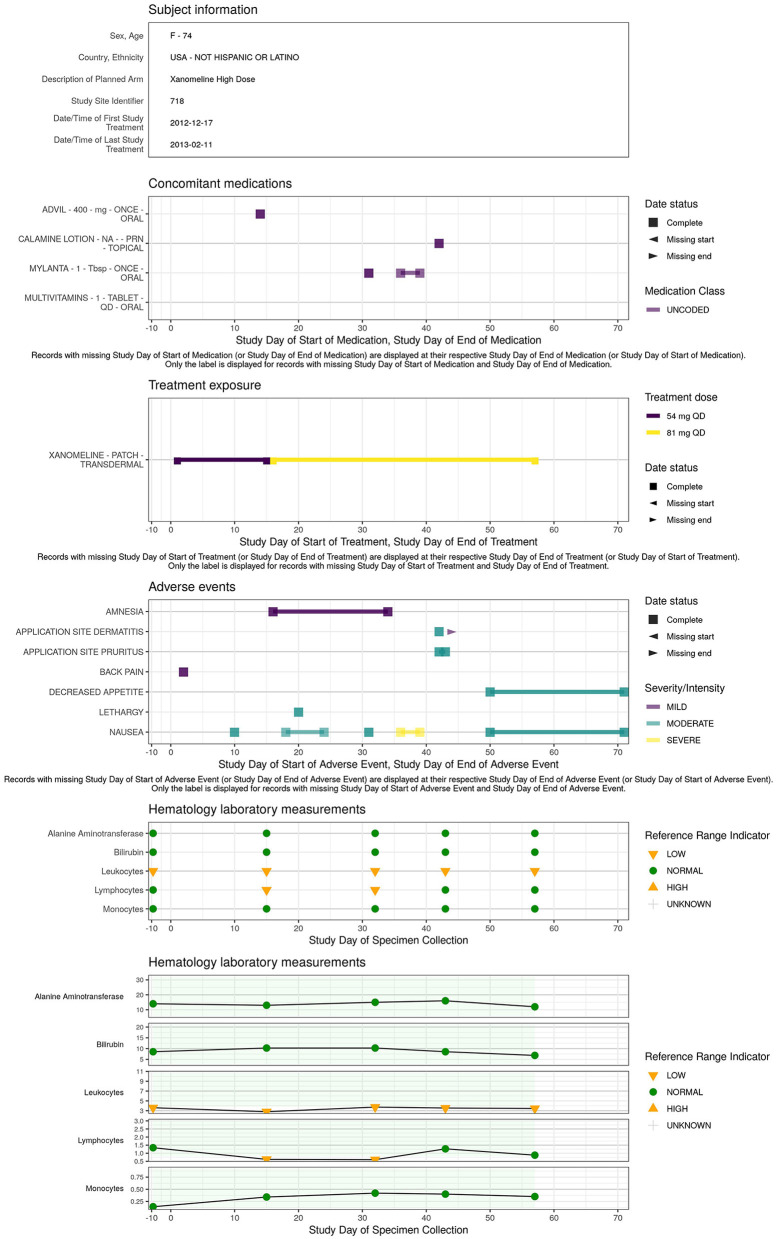
Example of patient level data aggregated across domains: patient profile.

A concise overview detailing the patient's demographics, medical background, exposure to treatments or concomitant medications, presence of adverse events, evolution of laboratory, ECG, vital signs parameters along the study timeline is essential for understanding the occurrence of adverse events or abnormal values detected in the earlier visualizations. The patient profile (Figure 8) displays an overview of all the clinical data for a specific patient.

### Efficacy

In a DMC setting, the tool can also support the reporting of efficacy data such that the medical monitor can evaluate the benefit-risk of the treatment. The tables and figures described above for safety monitoring (aggregated line plots in particular) can also be applied to compare the effect of the different treatment arms across patients.

### Reporting

At the biotechnology company, the tool has been considered critical for safety monitoring, although standard listings are available from other sources. The dynamic visualization, data extraction and drill-down capabilities of the tool are playing a critical role in the safety monitoring and as such need to be documented according to the EMA ICH6 (R2) (1).

EMA ICH6 (R2): *Results of monitoring activities should be documented in sufficient detail to allow verification of compliance with the monitoring plan*.

Thanks to the HTML format of the tool, the medical monitor is able to archive the report as part of the oversight documentation once the review is completed, facilitating GCP compliance and thereby allowing for appropriate traceability and accurate reporting, interpretation, and verification of the clinical trial-related information. The entire report can be exported, downloaded, and shared broadly and rapidly. On top of that, additional outputs in Excel, Word, or other standard formats can be created if preferred and filed in addition (see Figures 3, 5). Each report contains a unique identifier visible for the user, and is stored in a time-stamped location, for traceability and filing purposes.

## Discussion

The creation of interactive clinical data review reports with the clinDataReview software addresses the need for a timely and thorough view of the clinical data in an ongoing clinical trial while conforming to the strict regulatory requirements (reproducibility and traceability) regarding analysis workflows supporting decision making on patient data.

The open-source nature of the software means that it is ready to use, and no additional technology or license is required, making it a viable and cost-saving alternative compared to commercial solutions. As such, the report could be reproduced even by external parties or long after the end of the trial. With increasing development costs being a general concern in the industry, the tool substantially decreases the cost of medical monitoring and oversight while ensuring a standardized identification of safety risks within the trial early on.

There are no dependencies on external parties (besides on the data transfer level, if the clinical data base is outsourced) for the setup of the tool. The internal cross-functional effort means that the sponsor has a better grip on the data, can decide on the frequency of the periodic monitoring review and can follow-up the resolution of the identified data issues. Because the report can be easily used in-house, statisticians, programmers and/or medical monitors can review the data on an ongoing basis. This way the accuracy, integrity, and completeness, not only of the TLF data, can be followed-up from start to end of the trial.

For the medical monitors, the use of the report increases the efficiency and ease of the safety review leading to an improvement in its quality. The report can display any clinical trial data in one single place, going beyond the display of safety visualization only, including patient profiles, comparison of baseline demographics, medical history, concomitant medications, operational characteristics of the trial (e.g., patients-by-visit visualizations), and efficacy (if of interest). The medical monitoring task is greatly facilitated by eliminating the need to navigate through different clinical data bases (e.g., laboratory data base, electrocardiogram data base, eCRF…) to collect the data of interest. This makes the safety monitoring less error-prone and more efficient. The report contains a clear table of contents with hyperlinks to help the reviewer with locating and reporting the necessary information. The patient profile makes it more convenient to relate one parameter from one domain to another. Such a comprehensive and clearly structured report is deemed valuable for monitoring purposes (10).

The need to include data besides safety data (e.g., efficacy) in DMCs has recently still been recognized by the FDA DMC guideline (11). However, efficacy data is generally less standardized than safety data, which limits the possibility to use a standardized process. Including efficacy data might require more pre-processing to prepare the data set and extensive adjustments for each specific study.

As the report is consistent across studies, it requires little time for the medical monitors to get familiar with, compared to some of the other available medical monitoring software with a myriad of options and functionalities to select from. The standardization of the output ensures that every medical monitor will view the same kind of tables and graphs for all studies. This helps ensure that the core aspects are properly and consistently monitored, so the identification of safety signals depends less on how experienced the medical monitor is with the tool. Moreover, as the report is parametrized and modular, a specific visualization or table used for decision making can easily be traced back and reproduced.

The use of the tool for late phase clinical trials (especially phase 3), including many patients and/or visits pose few challenges. The time to create a report increases but has been minimized through parallelization of the report creation process (across chapters and/or patients). The size of the report increases with the number of patients, requiring more storage capacity (or a dedicated storage server), which can be reduced by focusing on a restricted set of patients with more safety risks (as patients with laboratory abnormalities or safety events). The browser rendering speed of some sections may be affected when many visualizations or subsections are present. This was tackled by chapter-specific exports into separated html files and by including buttons to select visualizations.

The set-up of this tool within the setting of a biotechnology company shows that R-based applications are viable in GxP settings, and a production system can be built to meet the appropriate regulatory standards. When relying on open-source software within a regulated computerized system, the primary challenge from a GxP standpoint lies in the development and maintenance of the tool-specific software. The validation of the entire tool using unit tests, as it is published on the Comprehensive R Archive Network (CRAN) Repository,^15^ was the result of a trade-off between the investment of time required for the validation of each component/functionality and its impact on the decision making resulting from the corresponding output. It is important that critical thinking is applied when determining the level of risk and mitigation strategies according to the quality risk management principles in the ICH Q9 guideline (12). As an example, the suite of unit tests covering the creation of the tables of summary statistics was deliberately more extensive than the code used to style the report (custom color palette or report styling sheet). The code base of the report can be extended with additional outputs (whether using custom or existing R packages), however their validation can be challenging and time consuming as the additional code would need to be validated in a similar risk-based manner.

The Continuous Integration/Continuous Development validation setup built has enabled us to automate the documentation trail needed when updates are made to the clinical data review software. All the required documentation—results of the tests and source code of the unit tests with specifications—can be generated in a matter of minutes (one validation form per package), leaving only the manual review from the Quality Assurance department to complete the validation. This setup can thus be viewed as a demonstration of Continuous Integration/Continuous Development validation in stark contrast to the efforts to re-validate solutions of yesteryear.

## Software availability

The example report and a demonstration of the tool are available on the website: medical-monitoring.openanalytics.io. The complete code specifications for this report are available in a github repository (link available on the website). The R packages adhere to the CRAN Repository Policy (see text footnote ^15^) and are available on the CRAN platform:

- clinDataReview: https://cran.r-project.org/package=clinDataReview
- inTextSummaryTable: https://cran.r-project.org/package=inTextSummaryTable
- patientProfilesVis: https://cran.r-project.org/package=patientProfilesVis.

## Data availability statement

Publicly available datasets were analyzed in this study. This data can be found here: https://github.com/phuse-org/phuse-scripts/tree/master/data/sdtm/cdiscpilot01.

## Author contributions

LC: Conceptualization, Software, Validation, Visualization, Writing – original draft, Writing – review & editing. MF: Conceptualization, Software, Supervision, Validation, Writing – original draft, Writing – review & editing. DK: Data curation, Software, Validation, Writing – review & editing. AD: Conceptualization, Software, Supervision, Validation, Writing – review & editing. MP: Software, Validation, Writing – review & editing. AF: Conceptualization, Writing – review & editing. CA: Conceptualization, Supervision, Validation, Writing – original draft, Writing – review & editing. PM: Conceptualization, Supervision, Writing – original draft, Writing – review & editing.

## Footnotes

^1^JReview. Integrated Clinical Systems, Inc. [Online]. Available online at: https://www.i-review.com/jreview.html.

^2^Spotfire. TIBCO Software [Online]. Available online at: https://www.spotfire.com/.

^3^Clinical Data Interchange Standards Consortium (CDISC). [Online]. Available online at: https://www.cdisc.org.

^4^CDISC. Analysis Data Model (ADaM) [Online]. Available online at: https://www.cdisc.org/standards/foundational/adam.

^5^CDIS. Standard Data Tabulation Format (SDTM) [Online]. Available online at: https://www.cdisc.org/standards/foundational/sdtm.

^6^YAML. YAML Ain't Markup Language [Online]. Available online at: https://yaml.org/.

^7^Kubernetes. [Online]. Available online at: https://kubernetes.io/.

^8^Amazon. Amazon Elastic Container Registry [Online]. Available online at: https://aws.amazon.com/ecr/.

^9^Amazon. AWS CodeCommit [Online]. Available online at: https://aws.amazon.com/codecommit/.

^10^Open Analytics NV. RDepot [Online]. Available online at: https://rdepot.io/.

^11^Sonatype Nexus repository [Online]. Available online at: https://www.sonatype.com/products/sonatype-nexus-repository.

^12^git [Online]. Available online at: https://git-scm.com/.

^13^SAS. Life Science Analytics Framework (LSAF). [Online]. Available online at: https://www.sas.com/en_au/software/life-science-analytics-framework.html.

^14^Jenkins [Online]. Available online at: https://www.jenkins.io/.

^15^CRAN Repository Maintainers. CRAN Repository Policy [Online]. Available online at: https://cran.r-project.org/web/packages/policies.html.
